# Controlling time-varying confounding in difference-in-differences studies using the time-varying treatments framework

**DOI:** 10.1007/s10742-023-00305-2

**Published:** 2023-03-16

**Authors:** Leslie Myint

**Affiliations:** Department of Mathematics, Statistics, and Computer Science, Macalester College, Saint Paul, MN USA

**Keywords:** Difference-in-differences, Time-varying treatments, Inverse probability weighting, Time-varying confounding, Treatment strategies

## Abstract

This article clarifies how the biostatistical literature on time-varying treatments (TVT) can provide tools for dealing with time-varying confounding in difference-in-differences (DiD) studies. I use a simulation study to compare the bias and standard error of inverse probability weighting estimators from the TVT framework, a DiD framework, and hybrid approaches that combine ideas from both frameworks. I simulated longitudinal data with treatment effect heterogeneity over multiple time points using linear and logistic models. Simulation settings looked at both time-invariant confounders and time-varying confounders affected by prior treatment. Estimators that combined ideas from both frameworks had lower bias than standard TVT and DiD estimators when assumptions were unmet. The TVT framework provides estimation tools that can complement DiD tools in a wide range of applied settings. It also provides alternate estimands for consideration in policy settings.

## Introduction

Difference-in-differences (DiD) designs are among the most popular tools for estimating causal effects of policy interventions. These designs are attractive for their wide applicability with longitudinal data and their ability to remove both measured and unmeasured confounding affecting the level (but not the trend) of the outcome under the parallel trends assumption.

Although parallel trends is a powerful identifying assumption, it is unlikely to hold exactly in many situations. Confounders that vary over time and/or have time-varying effects on the outcome can cause violations of the parallel trends assumption. This concern has led to a flurry of methods development under the assumption that parallel trends holds *conditionally* on covariates (see Roth et al. 2022 for a recent review). Methods that make a conditional parallel trends assumption prevailingly assume that control for pre-treatment covariates suffices. Researchers are often explicitly cautioned against controlling for post-treatment variables to avoid potential “post-treatment bias” (Rosenbaum 1984).

In fact, time-varying confounders that can be affected by treatment often *need* to be controlled for in order to validly estimate causal effects. (The phenomenon of confounders being affected by treatment has been described as “treatment-confounder feedback” in the biostatistics literature.) Zeldow and Hatfield (2021) demonstrate the bias that results in causal effect estimates when time-varying confounders affected by treatment are accounted for via regression and matching strategies, but none of the estimators they explore recovers the true causal effect in settings with treatment-confounder feedback.

Solutions for properly handling time-varying confounding under treatment-confounder feedback exist in the established biostatistical literature on the framework of time-varying treatments (TVT) in causal inference. Broadly, the TVT framework provides tools for estimating causal effects using longitudinal data. Of potential interest for DiD applications, it provides tools for thinking about evaluating the effects of treatments that can switch on and off and treatment rules that depend on covariates. Despite the common goals of the DiD and TVT literatures to study longitudinal data, the two frameworks have historically remained separate.

This article aims to clarify the parallels between the TVT and DiD literatures. I introduce notation, estimands, assumptions, and inverse probability weighting estimation procedures in the TVT framework and describe how these pieces relate to their DiD counterparts. I also perform a simulation study to compare the performance of TVT, DiD, and hybrid estimators. For applied researchers who might ordinarily consider a standard DiD analysis plan, this article provides alternate estimation strategies. I also discuss how the TVT framework allows for estimation of different causal estimands, which can be relevant for investigating a more diverse set of policy questions.

### Related literature

Researchers have begun to consider the issue of time-varying confounding control more deeply. Caetano et al. (2022) present estimators for standard DiD estimands (ATTs) under time-varying confounding with treatment-confounder feedback but work under the canonical two group, two time period design. They use conditional parallel trends as their main identifying assumption but do acknowledge an alternate identification assumption in the TVT framework. In this article, I show how standard DiD estimands can be estimated in the TVT framework in the multiple time period setting.

Shahn et al. (2022) present methods for ATT estimation using structural nested mean models (SNMMs). SNMMs are part of the family of “g methods” (generalized methods) for estimating causal effects of treatment strategies sustained over time. G-methods also include inverse probability weighting and the g-formula. These models are able to handle multiple time periods and treatment-confounder feedback. They are particularly suited to studying effect modification as they are able to explicitly quantify interactions between time-varying treatments and time-varying covariates (Naimi et al. 2017). Because the goal of this article is to clarify parallels between the TVT and DiD literatures, I focus on inverse probability weighting estimators which are common to both frameworks.

## The time-varying treatments framework

### Treatment strategies and potential outcome notation

The TVT framework is concerned with estimating the joint effect of a series of treatments on a final outcome. Let random variable $D_t$ denote treatment at time *t*, and let random vector ${\overline{D}}_T = (D_1, \ldots , D_T)$ denote a series of treatments through final time period *T*. This series of treatments is also called a *treatment history* or *treatment strategy*. Let $d_t$ and ${\overline{d}}_T$ be specific realizations of $D_t$ and ${\overline{D}}_T$ respectively. When referring to the entire treatment history through the final time point *T*, the *T* subscript can be dropped. (That is, ${\overline{D}}$ and ${\overline{d}}$ refer to the full treatment history/strategy.) For example, ${\overline{d}} = (0, \ldots , 0)$ is the “never-treat” strategy, and ${\overline{d}} = (1, \ldots , 1)$ is the “always-treat” strategy. These two strategies are examples of *static treatment strategies*: the series of treatments is a fixed sequence that does not depend on covariates. It is also possible to specify *dynamic treatment strategies* in which $D_t$ is a function of (possibly time-varying) covariates. However, this article will focus on static strategies, as static strategies have direct parallels with commonly used DiD estimands. (I will comment on the potentials for studying dynamic strategies in policy contexts in the Discussion.)

Let $Y_t({\overline{d}})$ denote the potential outcome at time *t* under treatment strategy ${\overline{d}}$. The overbar notation is also used to denote covariate histories. For a set of covariates ${\textbf{X}}_t = (X_{1t}, \ldots , X_{pt})$ measured at time *t*, let $\overline{{\textbf{X}}}_t$ denote the covariate history at time *t* consisting of the sequence of covariate values for each covariate from time 1 to time *t*.

### Causal estimands

In the TVT framework, causal effects are defined as contrasts of potential outcomes under arbitrary treatment strategies ${\overline{d}}$ and ${\overline{d}}^*$. While the average treatment effect across all units (ATE) $E[Y_t({\overline{d}}) - Y_t({\overline{d}}^*)]$ is most common in the TVT literature, average treatment effects on the treated (ATTs) may also be of interest.

Typical DiD estimands can be framed in terms of treatment strategy contrasts. In the canonical DiD setup where there are two groups of units (treated and control) measured over two time periods ($t=1,2$), two particular static treatment strategies are compared: ${\overline{d}} = (0, 0)$ (control group) and ${\overline{d}}^* = (0, 1)$ (treatment group). Generally, the causal estimand of interest is the average effect of treatment on the treated (ATT) in time period 2: $E[Y_2(0,1) - Y_2(0,0) \mid {\overline{D}} = (0,1)]$.

A common variation on the canonical DiD setup is the staggered rollout (multiple time period) design. In this design, there are multiple time periods in which units become and remain treated (reflecting the common viewpoint of treatment as an absorbing state). As in the canonical setting, ATT estimands are typically of interest and can be expressed in terms of treatment strategy contrasts. Here, treatment strategies can be indexed by the time period *g* in which units are first treated (referred to as a treatment “group”). Letting *T* be the total number of time periods, the static treatment strategies of interest are ${\overline{d}}^* = ({\textbf{0}}_{g-1}, {\textbf{1}}_{T-g+1})$ for $g = 1, \ldots , T$, where ${\textbf{0}}_a$ and ${\textbf{1}}_a$ are vectors of zeroes and ones repeated *a* times. The “never-treated” strategy ${\overline{d}} = {\textbf{0}}_T$ typically forms the reference strategy in causal contrasts. Let *G* denote the time at which a unit was first treated. The recent framework of Callaway and Sant’Anna (2021) (henceforth referred to as the “CS2021 framework”) centers the *group-time ATT* as the causal estimand of interest:

1 $$\begin{aligned} ATT(g,t) = E[Y_t({\overline{d}}^*) - Y_t({\textbf{0}}_T) \mid G = g], \qquad G = 2,\ldots ,T. \end{aligned}$$

The group-time ATT represents the causal effect for a given treatment timing group *g* in a given time period *t*. In the absence of anticipatory effects of treatment (potential outcomes being affected by future treatment), interest generally lies in estimating these group-time effects for $t \ge g$. (Researchers may be interested in checking for the absence of effects at times $t < g$ to check the plausibility of the parallel trends assumption.)

Note that the CS2021 framework does not estimate causal effects in the first time period. Units treated in the first time period are dropped from the analysis because, under the parallel trends assumption, untreated potential outcomes are never observed for these units: units treated in the first time period never have observed outcomes from time periods in which they were not treated. Under alternate (TVT framework) assumptions, causal effects for units treated in the first time period can be identified.

### Identification assumptions

In order to identify causal effects, the TVT framework relies on an assumption called sequential (conditional) exchangeability (also called ignorability), which states that potential outcomes are conditionally independent of treatment at a particular time *k* given prior treatment history ${\overline{D}}_{k-1}$ and covariate history $\overline{{\textbf{X}}}_k$:

2 $$\begin{aligned} Y_t({\overline{d}}) \perp \!\!\!\perp D_k \mid {\overline{D}}_{k-1} = {\overline{d}}_{k-1}, \overline{{\textbf{X}}}_k \qquad \text {for}\quad k = 1,\ldots ,T. \end{aligned}$$

We can think of sequential exchangeability as assuming a conditionally randomized experiment at each time point, where randomization depends on prior treatment and on covariates measured at prior and concurrent time points.

The identification of covariates needed for sequential exchangeability to hold has been aided by the use of causal graphs, which have a long history in biostatistics and epidemiology of clarifying the structural nature of biases in causal inference (Robins 2001). It is beyond the scope of this article to provide a detailed explanation of how to use causal graphs to identify suitable control variables, but I will provide a conceptual overview.

**Fig. 1:**
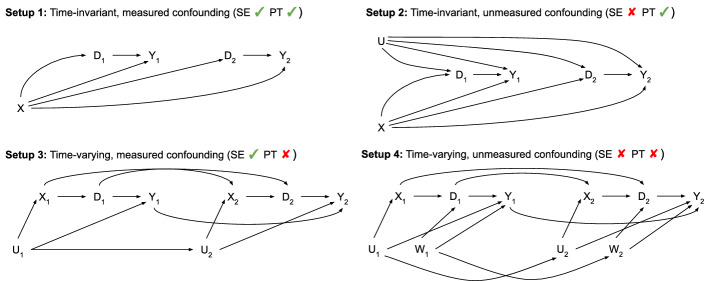
Causal graphs underlying simulation setup. Relationships between variables for the first two time points

A causal graph depicts the (assumed) causal relationships between variables in the system under study. Examples are shown in Fig. 1. Strong background knowledge should guide construction of the graph (Ferguson et al. 2020; Rodrigues et al. June 2022). Paths between the treatment and outcome variables have the potential to create statistical associations between them. These paths can be classified as either causal or noncausal.

Causal paths are directed paths (arrows flow in one direction) between the treatment and outcome and generate causal effects. Causal paths that would remain even after application of an intervention should be left intact.

Noncausal paths are undirected paths (arrows do not all flow in the same direction) between the treatment and outcome. These paths create spurious associations that need to be removed. Identification of variables that suffice to “block” spurious association flow (meet sequential exchangeability) can be identified from noncausal paths using a graphical technique called *d-separation* (Pearl 1995; Robins 2001) on a graphical object related to the causal graph: a *single-world intervention graph* (SWIG) (Richardson and Robins 2013). A SWIG is very similar to a causal graph but displays potential outcomes (instead of observed outcomes) and represents variables in both pre- and post-intervention forms simultaneously. Full details on the graphical procedure to identify variables needed to achieve sequential exchangeability are given in Chapter 19 of Hernán and Robins (2021). Once variables that suffice to achieve sequential exchangeability are identified from the SWIG, they are used in estimation procedures described in the next section.

In contrast to sequential exchangeability, DiD methods rely chiefly on the parallel trends assumption, which states that the outcomes for the treated and control units would have evolved in parallel had the treated units remained untreated:

3 $$\begin{aligned} E[Y_2(0,0) - Y_1(0,0) \mid {\overline{D}} = (0,1)] = E[Y_2(0,0) - Y_1(0,0) \mid {\overline{D}} = (0,0)] \end{aligned}$$

The conditional parallel trends assumption states that the parallel trends assumption holds conditionally on covariates ${\textbf{X}}$ (generally assumed to be time-invariant, often pre-treatment, covariates in the DiD literature):

4 $$\begin{aligned} E[Y_2(0,0) - Y_1(0,0) \mid {\overline{D}} = (0,1), {\textbf{X}}] = E[Y_2(0,0) - Y_1(0,0) \mid {\overline{D}} = (0,0), {\textbf{X}}] \end{aligned}$$

When there are multiple time periods, the conditional parallel trends assumption can be generalized to hold across all time windows with two time points (Callaway and Sant’Anna 2021).

The key difference between parallel trends and sequential exchangeability lies in the role of covariates. The sequential exchangeability assumption requires that all confounders be measured at each time point. Of note, this requires measuring both time-invariant and time-varying confounders. In this sense, sequential exchangeability is a stronger assumption than parallel trends because assuming parallel trends does not require measurement of confounders that only affect the level of the outcome and have constant composition between groups over time. The ability for parallel trends to handle time invariant unmeasured confounding is a major strength of the DiD design. However, economic and cultural factors that influence policy outcomes likely vary in composition over time, have time-varying effects on the outcome, and can be affected by treatment. In these cases, conditional parallel trends can be violated, but sequential exchangeability would allow for identification.

### Modeling and estimation

In the TVT framework, inverse probability weighting (IPW) can be used to estimate causal effects, similar to their use in the DiD literature (Abadie 2005; Callaway and Sant’Anna 2021; Sant’Anna and Zhao 2020; Stuart et al. 2014). Central to IPW approaches in both literatures is the estimation of the propensity score, the probability of receiving treatment conditional on an appropriate set of covariates. In the TVT framework, these covariates are those sufficient for achieving sequential exchangeability, and in the DiD framework, they are the covariates needed for conditional parallel trends to hold. The key difference between IPW approaches in the TVT and DiD frameworks is the form of the propensity score and/or the way in which weights are used (discussed further in Sect. 2.5).

#### ATE weights

In the TVT framework, stabilized IP weights for ATE estimands are given by

5 $$\begin{aligned} W^{ATE}({\overline{d}},{\textbf{X}}) = \prod _{t=1}^T \frac{P(D_t=d_t \mid {\overline{D}}_{t-1}={\overline{d}}_{t-1})}{P(D_t=d_t \mid {\overline{D}}_{t-1}={\overline{d}}_{t-1}, \overline{{\textbf{X}}}_{t})} \end{aligned}$$

Essentially, the denominator of the weights is a unit’s probability of receiving their particular treatment strategy conditional on their treatment and covariate history. Stabilized IP weights have lower variance than their unstabilized counterparts which use a numerator of 1 instead of $P(D_t=d_t \mid {\overline{D}}_{t-1}={\overline{d}}_{t-1})$.

Use of the ATE weights creates a pseudopopulation in which treatment indicators $D_t$ are independent of confounders ${\textbf{X}}$. As such, the mean potential outcome under any treatment strategy ${\overline{d}}$ is identified by the average outcome among units following this strategy in the weighted population (pseudopopulation). For example, $E[Y_t(0,1)]$ is identified by $E_{ps}[Y \mid D_1=0, D_2=1]$, where the latter expectation is with respect to the pseudopopulation created by the ATE weights. Essentially, we apply the weights and compute the mean outcome among the (reweighted) units following the treatment strategy of interest.

In practice, this is operationalized with regression models. For example, fitting the outcome regression model $E[Y_2 \mid d_1, d_2] = \beta _0 + \beta _1 d_1 + \beta _2 d_2$ using weighted least squares (using TVT ATE weights) provides estimates of the mean potential outcome at $t=2$ under any length 2 treatment strategy (e.g., $\beta _0$ represents the mean potental outcome for the (0, 0) strategy and $\beta _0 + \beta _1+\beta _2$ for the (1, 1) strategy). Outcome regression models fit with IP-weighted least squares are also called *marginal structural models* (MSMs).

#### ATT weights

Weights for ATT estimands (such as group-time ATTs) can be constructed in an analogous way to their construction in the single time point setting (treated units receiving a weight of 1 and control units receiving a weight equal to the conditional odds of treatment). However, in the multiple time period setting, we no longer have just one treated group; there are multiple groups of units who receive treatment (at different time points), and there are correspondingly different sets of ATT weights. Let ${\overline{d}}^*$ represent the treatment strategy for the treatment group of current interest, and let ${\overline{D}}$ represent the treatment strategy of the unit under consideration. The unnormalized ATT weights are given by:

6 $$\begin{aligned} W^{ATT}({\overline{d}}, {\textbf{X}}) = {\left\{ \begin{array}{ll} 1, &{} {\overline{D}} = {\overline{d}}^* \\ \prod _{t=1}^T \frac{P(D_t=d^*_t \mid {\overline{D}}_{t-1}={\overline{d}}^*_{t-1}, \overline{{\textbf{X}}}_{t})}{P(D_t=d_t \mid {\overline{D}}_{t-1}={\overline{d}}_{t-1}, \overline{{\textbf{X}}}_{t})}, &{} {\overline{D}} \ne {\overline{d}}^* \quad \text {and is a valid control} \\ 0, &{} {\overline{D}} \ne {\overline{d}}^* \quad \text {and is not a valid control} \end{array}\right. } \end{aligned}$$

Whether or not a unit is a “valid control” depends on (1) the time point *t* at which causal effects are desired and (2) the type of control units desired: never-treated units or not-yet-treated units. If using never-treated controls, only units following the ${\textbf{0}}_T$ treatment strategy are valid controls. If using not-yet-treated controls, valid controls comprise the never-treated units and the units who have not yet initiated treatment by time *t*.

It is useful to make explicit the parallels between single time point ATT weights and the ATT weights described here:

- In the single time point setting, treated units receive a weight of 1. Here, units following the treatment strategy of interest receive a weight of 1. The mean of $Y_t$ among these units estimates $E[Y_t({\overline{d}}^*) \mid {\overline{D}}={\overline{d}}^*]$.
- In the single time point setting, control units receive a weight equal to the conditional odds of treatment. That is, if $p({\textbf{X}})$ represents the propensity score, control units receive weights equal to $p({\textbf{X}})/(1-p({\textbf{X}}))$. The $1-p({\textbf{X}})$ in the denominator reweights control units to the full sample; it effectively simulates *all units* in the sample receiving the control condition. The $p({\textbf{X}})$ in the numerator scales the weight back down to have *just the treated units* receiving the control condition, allowing for estimation of the treatment effect for just the treated units. Here, the denominator of the weights for valid controls effectively simulates all units receiving each of the control treatment strategies. The numerator scales the weight back down to have *just the treated units* following the control strategies. The weighted mean of $Y_t$ among valid controls estimates $E[Y_t({\overline{0}}) \mid {\overline{D}}={\overline{d}}^*]$.

Normalized weights can be formed by normalizing the weights for control units to sum to the total number of treated units.

Similar to ATE weights, use of ATT weights creates a pseudopopulation in which treatment is independent of confounders. Unlike the ATE weights, the pseudopopulation created by the ATT weights cannot be used to estimate the marginal mean potential outcome under any treatment strategy; it can only be used to estimate the mean potential outcome under the treatment strategy for the treated group (${\overline{d}}^*$) and under the treatment strategies followed by valid controls *among the units who followed the treated strategy* ${\overline{d}}^*$.

Fitting MSMs at multiple time points parallels estimation goals in DiD settings–in particular, the estimation of group-time ATTs in the CS2021 framework. Estimating *ATT*(*g*, *t*) in the TVT (MSM) framework involves constructing TVT ATT weights for treatment strategy ${\overline{d}}^* = ({\textbf{0}}_{g-1}, {\textbf{1}}_{T-g+1})$ and fitting the MSM $E[Y_t] = \beta _0 + \beta _1 d_g$.

#### Weights for treatment initiation (absorbing treatments)

A slight modification of the ATE and ATT weights is needed when estimating the effect of *initiating* a treatment. This detail is relevant in DiD settings that consider the effect of an absorbing treatment. Once a unit is treated (treatment “switches on”), the conditional probability of treatment given treatment and covariate history is 1. That is, if a unit is first treated at time *g*, the terms in the product of (5) and (6) become 1 for $t > g$. For example, the treatment strategy ${\overline{d}} = (0,1,1,1)$ in a 4-time period setting is effectively treated as the strategy ${\overline{d}} = (0,1)$ in constructing IP weights. In the remainder of this article, I will refer to the weights described above as TVT IP weights.

### Comparison of TVT and DiD approaches to IPW

Given that IPW has a long history in the DiD literature (Abadie 2005; Callaway and Sant’Anna 2021; Sant’Anna and Zhao 2020; Stuart et al. 2014), it is useful to compare the details of IPW implementation in these frameworks and in the TVT framework.

The form of the TVT IP weights clarifies why these weights can handle time-varying confounding. Recall that the sequential exchangeability assumption can be interpreted as an assumption of a conditionally randomized experiment at each time point. Each term in the product form of the TVT IP weights reflects randomization at a single time point, where randomization can be influenced by any type of confounder: time-fixed or time-varying. Thus, application of the TVT IP weights creates a pseudopopulation in which treatment is marginally independent of the confounders and past treatment at each time point. This allows for direct comparison of mean outcomes under different treatment strategies in the weighted sample. In terms of the causal graph, IP weighting removes arrows from confounders to treatment at each time point, thus disabling noncausal paths.

In contrast, IPW strategies in the DiD literature have primarily used time-invariant (often pre-treatment) covariates in propensity score estimation. Thus, weighting creates a conditionally randomized experiment at the first time point, but not at subsequent time points and is thus unable to handle time-varying confounding.

Another key difference between TVT and DiD strategies for IPW is the quantity that is weighted. Abadie (2005); Callaway and Sant’Anna (2021); Sant’Anna and Zhao (2020) provide estimators that weight outcome *change scores* (e.g., $Y_{t=2} - Y_{t=1}$) in addition to estimators that weight the outcomes themselves. The use of change score estimators has efficiency benefits (Sant’Anna and Zhao 2020) but requires the availability of panel data. Using the change score has the benefit of removing the effect of certain types of unmeasured confounders (confounders that do not evolve differently over time in the treatment and control groups and do not have a time-varying effect on the outcome) Because the TVT literature has focused on treatment effects at a single final time point, this useful feature of the change score seems to have been overlooked in TVT methodology. It is natural to consider combining TVT IP weighting with construction of change scores. I explore this in my simulation studies.

## Methods

I conducted a simulation study to compare the performance of TVT IP weighting (Hernán and Robins 2021), DiD IP weighting, and estimation strategies fusing ideas from both frameworks. For DiD methods, I focused on the CS2021 framework because it handles most of the major concerns in the recent literature on DiD methodology (multiple time periods, conditional parallel trends, avoiding problems with two-way fixed effects regressions). Simulation code is available on GitHub (https://github.com/lmyint/tvt_did).

### Data generating mechanisms

Simulated panel datasets were generated from four structural equation models (Table 1). The causal graphs associated with these models are shown in Fig. 1. *U* and *W* are unmeasured confounders, *D* is treatment, *X* is an observed proxy for *U* that influences treatment, and *Y* is the outcome. These four data-generating mechanisms represent situations with time-invariant (Setups 1 and 2) and time-varying confounding (Setups 3 and 4). They also represent situations in which all confounders are measured or have measured proxies (Setups 1 and 3) and in which there are unmeasured confounders (Setups 2 and 4). All four setups exhibit treatment effect heterogeneity (due to an interaction between $D_t$ and *X* in Setups 1 and 2 and due to an interaction between $D_t$ and $U_t$ in Setups 3 and 4).

In Setup 1, both sequential exchangeability and parallel trends given *X* hold. Relative to Setup 1, Setup 2 contains an additional time-invariant unmeasured confounder *U*. Here, parallel trends holds, but *U* causes sequential exchangeability to be violated. In Setup 3, time-varying confounder *U* and its time-varying proxy *X* cause a violation of parallel trends, but sequential exchangeability given *X* holds. Relative to Setup 3, Setup 4 contains an additional unmeasured confounder *W* that causes sequential exchangeability to be violated.

With the exception of *U* and *X* in Setups 1 and 2, all variables are time-varying and were simulated over 4 time points. (When simulating the variables at time $t=1$, variables at time $t-1$ were dropped from the structural equations.) All variables were continuous except for treatment, which was binary. The structural equation for treatment gives the log odds of treatment. In correspondence with much of the DiD literature, treatment was simulated to be an absorbing state; once a unit was treated, it remained treated for the remaining time periods. (The structural equation for *D* was ignored for a unit once it had been treated.)

The strength of confounding was varied by scaling the coefficients on *X*, *U*, and *W* in the structural equations for treatment *D* and by scaling the coefficients on *X*, *U*, and *W* in the structural equations for outcome *Y* (parameter *m* in Table 1). In each of these settings, a full population of one million units was simulated. (Below I will refer to this as the “natural” population, as opposed to the population under a given treatment strategy.) From this population, I drew 100 datasets of sample size 3000.

To obtain the true values of causal effects (both ATEs and group-time ATTs), I used the structural equations to simulate the full population under intervention. Each unit’s potential outcome under a given treatment strategy ${\overline{d}} = (d_1, d_2, d_3, d_4)$ was computed by ignoring the structural equation for *D*, replacing $D_t$ with $d_t$ in the other structural equations, and initializing the system with the original values of all exogenous variables (noise terms; *U* and *X* in Setups 1 and 2; $U_1$ and $W_1$ in Setups 3 and 4). I computed true ATEs using the overall mean potential outcomes under different treatment strategies. I computed true group-time ATTs by using the mean potential outcomes among units following a given treatment strategy in the natural population.

**Table 1:** Structural equations for simulation setup. Error terms $\varepsilon _{\cdot }$ and $\varepsilon _{\cdot t}$ are independent and follow a standard normal distribution. Parameter $m \in \{1,2,3\}$ is used to vary the strength of confounding

Setup 1: Time-invariant, measured	Setup 2: Time-invariant, unmeasured
	$$U = \varepsilon _U$$
$X = \varepsilon _X$	$X = \varepsilon _X$
$D_t = -1 - 0.5 m X$	$D_t = -1 - 0.5 m X - 0.5 m U$
$Y_t = 0.5 m X + 0.5 D_{t} + 0.1 X D_{t} + \varepsilon _{Yt}$	$Y_t = 0.5 m X + 0.5 m U + 0.5 D_{t} + 0.1 X D_{t} + \varepsilon _{Yt}$

Setup 3: Time-varying, measured	Setup 4: Time-varying, unmeasured
	$$W_t = 0.5W_{t-1} + \varepsilon _{Wt}$$
$U_t = 0.5U_{t-1} + \varepsilon _{Ut}$	$U_t = 0.5U_{t-1} + \varepsilon _{Ut}$
$X_t = 0.5 U_{t} + 0.5 D_{t-1} + \varepsilon _{Xt}$	$X_t = 0.5 U_{t} + 0.5 D_{t-1} + \varepsilon _{Xt}$
$D_t = -1 - 0.5 m X_{t} - 0.5 m X_{t-1} + 0.5 D_{t-1}$	$D_t = -1 - 0.5 m X_{t} - 0.5 m X_{t-1} - 0.5 m W_{t} + 0.5 D_{t-1}$
$Y_t = 0.5\,m U_{t} + 0.5 D_{t} + 0.5 Y_{t-1} + 0.1 U_{t} D_{t} + \varepsilon _{Yt}$	$Y_t = 0.5\,m U_{t} + 0.5\,m W_{t} + 0.5 D_{t} + 0.5 Y_{t-1} + 0.1 U_{t} D_{t} + \varepsilon _{Yt}$

### Effect estimation

#### Time-varying treatment framework

Under all data-generating setups, I assume that time-varying covariate *X* suffices to achieve sequential exchangeability. This assumption holds in Setups 1 and 3 but not in Setups 2 and 4.

I first constructed stabilized inverse probability ATE weights as detailed in Sect. 2.4 by fitting logistic regression models for treatment at each time point. The ATE weights were used to fit the MSMs below to estimate concurrent and dynamic (lagged) treatment effects. Because these models describe the average potential outcome under any static treatment strategy at each time point, they can be used to estimate any static treatment effect. All model coefficients were causal estimands of interest. The $\beta _{t0}$ coefficients represent average potential outcomes at time *t* when remaining untreated up to time *t*. Under an absorbing treatment setup, the $\beta _{td}$ coefficients represent a contrast of strategies $({\textbf{0}}_{d-1}, {\textbf{1}}_{5-d})$ and $({\textbf{0}}_{d}, {\textbf{1}}_{4-d})$ at time *t*.

7 $$\begin{aligned} E[Y_1]&= \beta _{10} + \beta _{11} D_1 \nonumber \\ E[Y_2]&= \beta _{20} + \beta _{21} D_1 + \beta _{22} D_2 \nonumber \\ E[Y_3]&= \beta _{30} + \beta _{31} D_1 + \beta _{32} D_2 + \beta _{33} D_3 \nonumber \\ E[Y_4]&= \beta _{40} + \beta _{41} D_1 + \beta _{42} D_2 + \beta _{43} D_3 + \beta _{44} D_4 \end{aligned}$$

I also used the TVT IP weighting framework to compute ATT weights for estimating group-time ATTs, as described in Sect. 2.4. I used two choices for the control group: (1) units who were never treated and (2) units who were not yet treated by the time point of interest. In addition to weighting the outcomes directly, I used TVT ATT weights to weight change scores (a “hybrid” estimator). Specifically, I formed the change score $\Delta _{g,t} = Y_t - Y_{g-1}$ and fit the model $E[\Delta _{g,t}] = \alpha _0 + \alpha _{g,t} D_g$ using TVT ATT weights. I used $\alpha _{g,t}$ as an estimate of *ATT*(*g*, *t*).

Use of the TVT IP weights (for both ATE and ATT estimands) effectively removes the $X \rightarrow D$ arrows in Fig. 1. While this seems to disable a causal path ($D_t \rightarrow X_{t+1} \rightarrow D_{t+1} \rightarrow Y_{t+1}$), this is not a path that would remain after application of an intervention. An intervention would remove the influence of any variables governing treatment decisions: the $X_{t+1} \rightarrow D_{t+1}$ arrow would not exist, and the aforementioned directed path would not exist.

#### Group-time average treatment effect framework

The did R package implements the CS2021 estimators. I used this package to estimate group-time ATTs and varied the construction of estimators along three axes: (1) the choice of control group (never treated, not yet treated), (2) covariate adjustment (adjusted and unadjusted for *X*), and (3) weighting scheme (unweighted and weighted using TVT ATE weights). Regarding covariate adjustment, while the CS2021 framework does not allow for time-varying covariates, *X* was still partially adjusted for in that only its values at the first time point were used as pre-treatment covariates. Regarding weights, CS2021 estimators are unweighted by default but can optionally incorporate sampling weights. As part of a hybrid approach, I investigated the use of TVT-ATE weights in these estimators (rather than TVT-ATT weights) to understand whether this could rectify the problems caused by time-varying confounding. The rationale for using ATE rather than ATT weights was twofold. First, using ATE weights is simpler in that a single set of weights can be used. In contrast, using ATT weights would require a different set of weights for each group-time ATT. Second, application of the ATE weights creates a pseudopopulation free from confounding by *X*. I wanted to understand whether application of CS2021 estimators in this pseudopopulation could recover the true group-time ATTs.

## Results

### ATE estimands

**Fig. 2:**
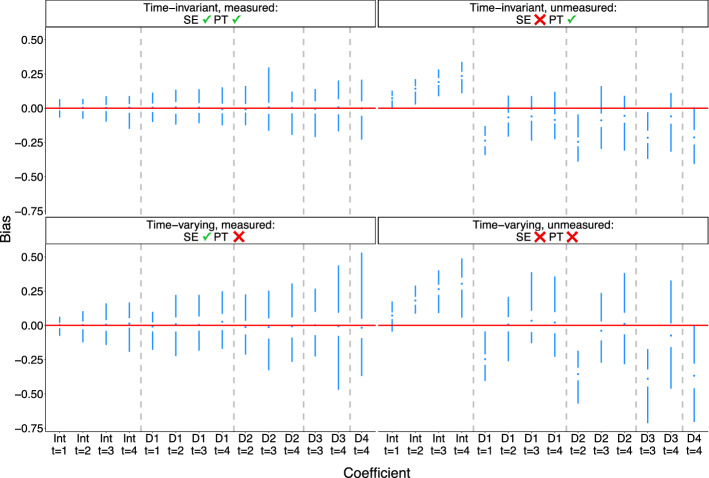
Bias of estimates of marginal structural model coefficients. Results are shown for the lowest level of confounding strength ($m=1$ in the structural equations of Table 1). SE = sequential exchangeability. PT = parallel trends

**Fig. 3:**
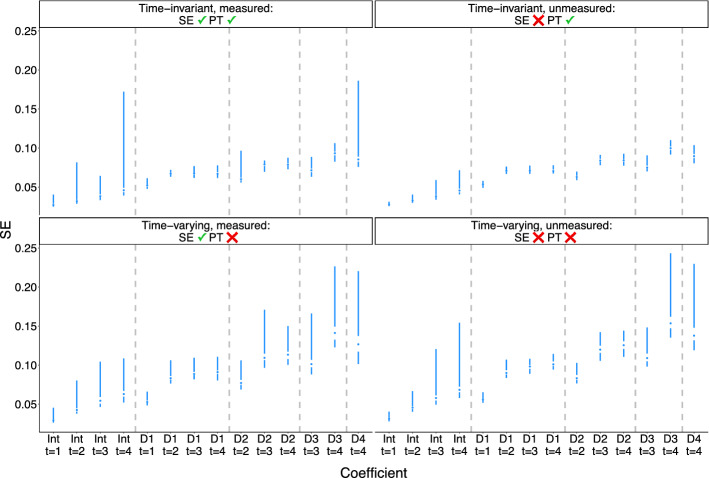
Standard error of estimates of marginal structural model coefficients. Same context as Fig. 2

Figures 2 and 3 show the bias and standard error of MSM coefficient (ATE) estimates using TVT estimators. As expected, estimators are unbiased when sequential exchangeability is met and biased otherwise. Standard errors are higher for treatment effects at later time periods and at time points further past the point of initial treatment (higher lags).

### Group-time ATT estimands

**Fig. 4:**
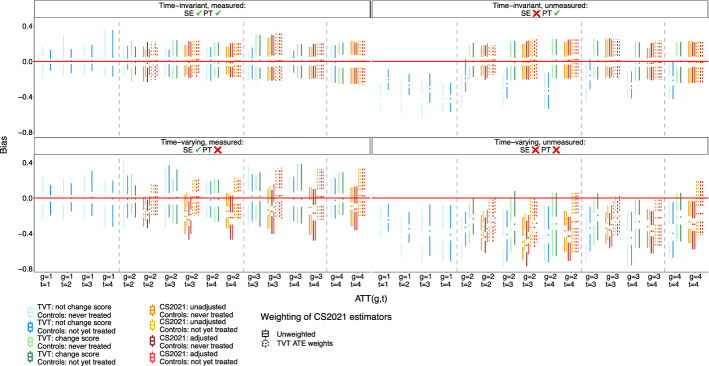
Bias of group-time ATT estimates from TVT and CS2021 estimators. Results are shown for the lowest level of confounding strength ($m=1$ in the structural equations of Table 1). SE = sequential exchangeability. PT = parallel trends

**Fig. 5:**
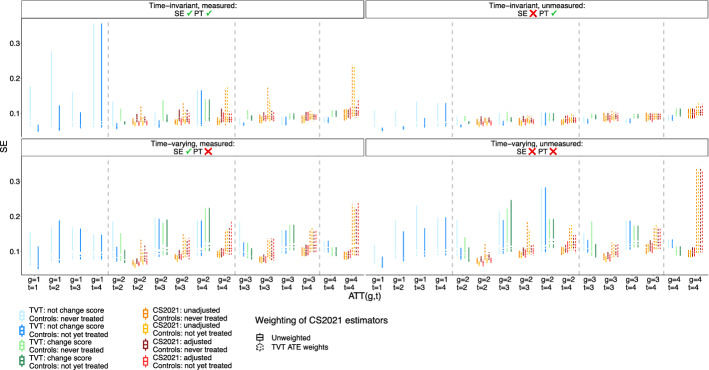
Standard error of group-time ATT estimates from TVT and CS2021 estimators. Same context as Fig. 4

Figures 4 and 5 show the bias and standard error of group-time ATT estimates using TVT, CS2021, and hybrid estimators. Under the simplest case of time-invariant measured confounding, all estimators are unbiased. When a time-invariant unmeasured confounder is added, TVT estimators that weight the outcome are biased, but estimators that weight the change score are unbiased.

When confounders are time-varying and measured (or measured proxies suffice to control confounding under sequential exchangeability), parallel trends is violated, and thus, unweighted CS2021 estimators are biased. However, CS2021 estimators that use TVT ATE weights become unbiased. When there is uncontrolled, unmeasured, time-varying confounding, all estimators are biased. However, TVT-change score and TVT-weighted CS2021 estimators have comparable bias, and this bias is lower than those of TVT-outcome and unweighted CS2021 estimators.

Across all data-generating setups, bias and standard error increase at greater lags. Further, standard errors for TVT estimators and TVT-weighted CS2021 estimators are higher than for unweighted CS2021 estimators. Estimates based on a not-yet-treated control group are less biased than estimates based on a never-treated control group.

**Fig. 6:**
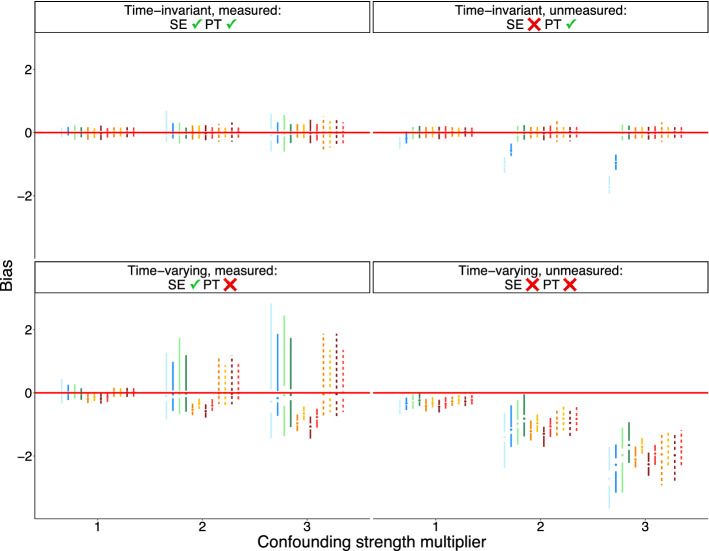
Bias for $ATT(g=2,t=2)$ as a function of confounding strength. Same plot legend from Figs. 4 and 5

**Fig. 7:**
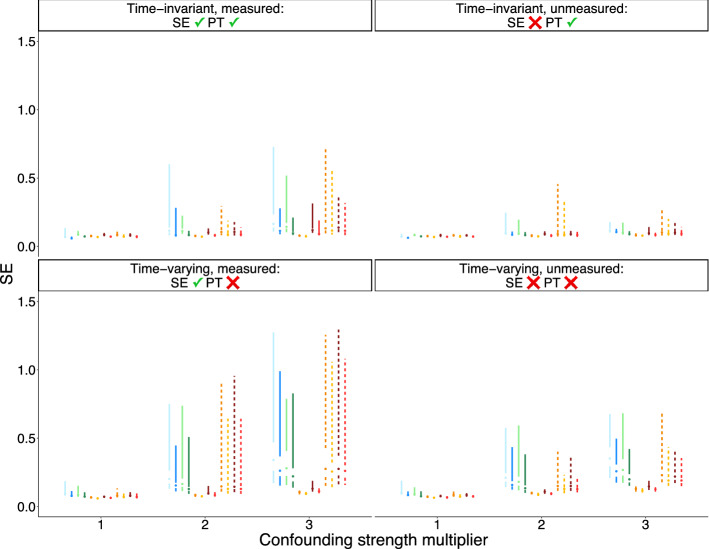
Standard error for $ATT(g=2,t=2)$ as a function of confounding strength. Same plot legend from Figs. 4 and 5

Increasing strength of confounding exacerbated bias under all data setups and resulted in larger and more variable standard errors (Figs. 6 and 7).

## Discussion

This article contributes to the DiD literature by clarifying synergies between the DiD and TVT frameworks.

My simulations looked at all combinations of sequential exchangeability and parallel trends holding and were thus able to highlight the power of fusing idea from both frameworks. When a framework’s assumptions were unmet, its corresponding estimators were biased, but using an idea from the other framework led to improved performance. When sequential exchangeability was unmet but parallel trends held, adapting TVT estimators to weight change scores (change scores being more common in the DiD literature) rather than outcomes improved performance. When parallel trends was unmet but sequential exchangeability held, adapting DiD estimators by incorporating TVT weights improved performance. When both assumptions were unmet, hybrid estimators were less biased than standard estimators.

An advantage of the TVT framework is that under the sequential exchangeability assumption, it is possible to estimate effects for those treated in group 1 (when weighting outcomes rather than change scores). Under a parallel trends framework, units treated in the first time period would be discarded from the analysis.

While this study has focused on confounding control in longitudinal data settings, integrating the TVT framework with the DiD literature can be useful for other reasons. For one, the TVT literature provides a natural framework for estimating the effects of treatments that turn and off, a growing area of interest in the DiD literature (Chaisemartin and D’Haultfoeuille 2022). The methods discussed in this article directly apply to static treatment strategies such as ${\overline{d}} = (0,1,0,1)$ that represent a treatment switching on and off at regular intervals.

Further, the TVT framework provides estimators for dynamic treatment strategies, in which decisions to treat at each time point are functions of past treatment and covariates. Causal effects of dynamic treatment strategies are likely of substantial interest in policy settings where implementation details can vary considerably. The IP weighting approaches discussed in this article have been adapted to estimate the effects of dynamic strategies (Hernán et al. 2006) and could facilitate more detailed examinations of policy effects in studies where DiD is normally applied.

The TVT framework is not without limitations. In contrast to CS2021 estimators, TVT estimators had larger and more variable standard errors. Although TVT estimators had lower bias, their decreased power is an important tradeoff to consider. In this regard, it may be more prudent to use TVT estimators for concurrent rather than lagged causal effects: standard errors for TVT estimators were lowest and least variable for concurrent causal effects.

Doubly robust estimators have become an important part of causal inference to deal with the potential for model misspecification. While doubly robust methods for studying static treatment strategies exist (Hernán and Robins 2021), they do not seem to have user-friendly software implementations as is the case for DiD estimators (e.g., the did package for doubly-robust CS2021 estimators).

While sensitivity analyses for unmeasured confounding have seen substantial consideration in single time-point settings, the corresponding literature in the TVT framework has not been actively developed. Practitioners can still repeat analyses for multiple sets of potential confounders as a form of sensitivity analysis, but tools that help quantify the strength of unmeasured confounding needed to qualitatively change results (such as the E-value (VanderWeele and Ding 2017)) do not yet exist in the TVT framework. On the other hand, the DiD literature has an active area of research in this area (Manski and Pepper 2018; Rambachan and Roth 2022).

The relationship between the hybrid approaches investigated in this paper and alternate identifying assumptions could be an interesting area of further research. In particular, Shahn et al. (2022) make a time-varying conditional parallel trends assumption that appears to be in the same spirit as TVT estimators that weight change scores. Clarifying the connections between sequential exchangeability and parallel trends in their standard and alternate (hybrid) forms would be illuminating.

The TVT framework should be useful to practitioners accustomed to DiD methods. It contributes tools that can be used on top of existing DiD estimators (applying TVT weights as sampling weights) and that can be combined with DiD ideas (change scores) to improve estimation accuracy of causal effects. It also highlights additional policy questions that can be investigated through alternative causal estimands.

## Data Availability

Code to reproduce the results in this manuscript is available on GitHub (https://github.com/lmyint/tvt_did).
